# Prenatal 17‐Alpha‐Hydroxyprogesterone Caproate Exposure in Twins and Childhood Outcomes: 14‐Year Follow‐Up of a Randomised Trial

**DOI:** 10.1111/1471-0528.70223

**Published:** 2026-03-23

**Authors:** Emilie V. J. van Limburg Stirum, Marjon A. de Boer, A. C. J. Ravelli, Rik van Eekelen, Aleid G. Leemhuis, Martijn J. J. Finken, Cornelieke S. H. Aarnoudse‐Moens, Anneloes L. van Baar, Noor E. Simons, Rebecca C. Painter, Arianne C. Lim, Ben W. Mol, Eva Pajkrt, Martijn A. Oudijk, Janneke van 't Hooft

**Affiliations:** ^1^ Department of Obstetrics and Gynaecology Amsterdam UMC Location University of Amsterdam Amsterdam the Netherlands; ^2^ Amsterdam Reproduction and Development Amsterdam the Netherlands; ^3^ Department of Obstetrics and Gynaecology Amsterdam UMC Location Vrije Universiteit Amsterdam Amsterdam the Netherlands; ^4^ Department of Epidemiology and Biostatistics Amsterdam UMC Location VUmc Amsterdam the Netherlands; ^5^ Department of Neonatology and Paediatrics Emma Children's Hospital, Amsterdam UMC, University of Amsterdam Amsterdam the Netherlands; ^6^ Department of Paediatric Endocrinology Emma Children's Hospital, Amsterdam UMC, Vrije Universiteit Amsterdam Amsterdam the Netherlands; ^7^ Amsterdam UMC Location University of Amsterdam, Emma Children's Hospital, Child and Adolescent Psychiatry and Psychosocial Care Amsterdam the Netherlands; ^8^ Child and Adolescent Studies Utrecht University Utrecht the Netherlands; ^9^ Department of Obstetrics and Gynaecology Sophia Women's and Children's Hospital, Erasmus MC Rotterdam the Netherlands; ^10^ Department of Gynaecology and Obstetrics, GROW School of Oncology and Developmental Biology Maastricht University Medical Centre Maastricht the Netherlands; ^11^ Department of Obstetrics and Gynecology Monash University Clayton Victoria Australia

**Keywords:** child development, long‐term follow‐up, preterm birth, progesterone

## Abstract

**Objective:**

Long‐term child outcomes after intra‐uterine exposure to 17‐alpha‐hydroxyprogesterone caproate (17‐OHPC) versus placebo.

**Design:**

Follow‐up of the AMPHIA randomised controlled trial assessing neonatal outcomes after weekly 17‐OHPC injections compared to placebo in multiple pregnancies.

**Setting:**

Multicentre.

**Population or Sample:**

Children born in the AMPHIA study.

**Methods:**

Children were assessed at 11–14 years of age by record linkage and through self‐reported, parental and teacher questionnaires. Mean differences or odds ratios (OR) with 95% confidence intervals (95% CI) were calculated with cluster robust standard errors for twins and adjustment for confounders using inverse probability weighting (IPW).

**Main Outcome Measures:**

Child mortality, educational attainment, cognition, behaviour, gender identity, general health, and a composite of abnormal development.

**Results:**

Up to 14 years, 60/1356 children had died (*n* = 24 17‐OHPC and *n* = 36 placebo (OR 0.75 95% CI 0.36–1.53)). Record linkage was completed for 1027/1296 (79%) surviving children (*n* = 517 17‐OHPC and *n* = 510 placebo). No significant difference was found regarding educational attainment. Detailed developmental outcomes through questionnaires were collected for 303/1296 (23%) surviving children (*n* = 159 17‐OHPC and *n* = 144 placebo). No differences in cognition, behaviour, gender identity, and general health up to 14 years of age were found between both groups. The composite of abnormal development did not differ between 17‐OHPC (23.3%) and placebo (20.1%) groups (OR 1.31 95% CI 0.66–2.56).

**Conclusion:**

In offspring of women with a multiple pregnancy, the use of 17‐OHPC in pregnancy is unlikely to provide positive or negative effects on child outcome related to mortality, educational attainment, cognition and behaviour.

## 1 Introduction

Endogenous progesterone levels increase during the course of pregnancy and have several functions. First, it seems to play a role in fetal brain development and maturation [1, 2, 3, 4, 5]. Progesterone and its metabolite allopregnanolone have immune‐ and neuroprotective properties [1, 6]. They also influence neurotransmitter pathways, for example the mesocortex dopamine pathway involved in executive functioning [7, 8, 9, 10]. Moreover, the distribution of progesterone receptors across the hypothalamus differs between sexes, suggesting that progesterone might play a role in gender identity [11]. Second, progesterone has an essential role in maintaining pregnancy by a variety of actions, including inhibition of the oxytocin receptors and dampening of inflammation [8, 12]. Progestagens are therefore widely used in pregnant women at risk for preterm birth [13].

Progestagens such as 17‐alpha‐hydroxyprogesterone caproate (17‐OHPC) enter the fetal compartment where it crosses the fetal brain barrier [14]. As the pharmacodynamics of synthetic 17‐OHPC is not directly comparable to that of natural progesterone, it is conceivable that synthetic 17‐OHPC could interfere with the levels of progesterone and its (neuroprotective) metabolites in the fetus [1]. This implies that maternal use of 17‐OHPC in pregnancy could affect the developing brain of the fetus and, consequently, child development later in life.

Several studies have examined the influence of progestagen exposure on outcome of the offspring. In a meta‐analysis, based on five randomised controlled trials (RCTs) evaluating the long‐term effects of prenatal (vaginally administered) progesterone exposure on child development, no harmful or beneficial effects on child development up to the age of 8 years were reported [15]. However, follow‐up beyond 8 years of age has not been undertaken, despite the fact that cognitive and behavioural functioning continue to develop [16]. Therefore, we aim to evaluate the effect of prenatal exposure to progestagen treatment versus placebo on child development up to early adolescence.

## 2 Methods

We performed a follow‐up of the AMPHIA trial (17‐Alpha hydroxyprogesterone caproate in Multiple pregnancies to Prevent Handicapped InfAnts, ISRCTN 40512715). In this multicentre double‐blind placebo‐controlled randomised trial, women with a multiple pregnancy received weekly intramuscular injections of 250 mg 17‐OHPC (*n* = 336; 681 children) or placebo (*n* = 335; 674 children) to prevent adverse neonatal outcome by reducing the preterm birth rate. Details and results of the original trial are described elsewhere [17]. The follow‐up study was approved by the Medical Research Ethics Committee of the Amsterdam University Medical Center (Amsterdam UMC), location AMC (W20_234#20.268) and the protocol was published in a peer reviewed journal [18]. The manuscript is reported following Strengthening the Reporting of Observational Studies in Epidemiology (STROBE) guidelines [19].

### 2.1 Follow‐Up Assessment

Follow‐up assessment was performed through two different strategies. First, by record linkage with the Dutch Personal Records Database and education registries of Statistics Netherlands (CBS) (Project 9960). Second, by questionnaires that were completed by children, parents, and teachers. All women and their children born within the AMPHIA trial were eligible for participation.

In the original trial, participants gave consent to be approached for a follow‐up study. Medical records and the Dutch Personal Records Database were checked for the possible occurrence of maternal or child death, as well as to identify mail addresses of participants. Participants who, according to our data, had lost all children of the multiple pregnancy were not approached for assessment by questionnaires, yet their information was included in follow‐up analyses. In case of maternal death, current caregiver(s) were asked to participate together with the child(ren).

Between September 2020 and December 2022, 48 contributing hospitals of the AMPHIA trial sent information letters to the participants of the original trial and their child(ren). Participants who were interested in participating in the follow‐up study could send their contact details to the research team in the Amsterdam UMC and were then contacted by phone or email for the informed consent procedure. Parent(s)/caregiver(s) and child(ren) above 12 years of age had to sign their own informed consent form. Online questionnaires were sent in Castor EDC [20] when both children were between 11 and 14 years of age and attending high school. The questionnaire for parents took approximately 30 min per child to complete. The child and teacher's questionnaires required 10–15 min to complete. If questionnaires remained unfinished, participants received a reminder by email within 4 weeks.

In the original trial, participants and researchers were blinded for treatment allocation. Participants had the option to request information about their treatment allocation after publication of the trial results, but only a small proportion of participants filed a request. Before the start of the follow‐up, we could not identify which participants received information about their allocation. If participants were not aware of their treatment allocation, unblinding was offered after completion of the questionnaires or upon request. Researchers involved in data collection were blinded for treatment allocation in this follow‐up.

### 2.2 Outcome Measures

The main outcomes of this study included child mortality, educational attainment, cognition, and behaviour. Other outcomes included gender identity and general health of the children.

#### 2.2.1 Mortality

Child mortality was defined as perinatal death (from 16 weeks of gestation up to discharge from the hospital) or child death up to 14 years of age. Medical records and the Dutch Personal Records Database were checked for the occurrence of death for all children born to mothers in the original AMPHIA trial.

#### 2.2.2 Educational Attainment

Educational attainment was assessed by a record linkage with the CBS with two different education registries. This linkage provided data on children attending a special needs education school for 1 year or more at primary school age. This is a compulsory registry. We also collected data of the most frequently used standardised nationwide test taken at the end of primary school (Centraal Instituut voor Toets Ontwikkeling [CITO]) and compared the mean scores between groups. The CITO test is conducted when the child is 11 or 12 years of age in the final class of primary school and comprises 240 multiple‐choice items. It assesses various cognitive abilities (e.g., language and mathematics) and results in a composite score. This total score is usually presented as a standardised score between 501 and 550 (mean 535, SD 10). This standardised score is a guideline for recommendations concerning the type of secondary school [21]. The CITO test was not carried out in 2020 because of the COVID pandemic and, therefore, CITO scores were collected for a subsample of the AMPHIA population. Finally, teacher's recommendations at age 12 for the level of secondary school, which provides guidance for the appropriate level for the children into vocational or higher secondary education, were collected for the AMPHIA population.

Additionally, questions about the current educational level, whether the child had repeated a class, and whether the child needed additional help inside or outside the classroom were filled out by the parents in the general health questionnaire. Secondary school teachers were also asked about the child's need for extra help inside or outside the classroom.

#### 2.2.3 Cognition

Cognition was assessed by the Dutch version of the Behaviour Rating Inventory of Executive Function (BRIEF) screener and was filled out by the children (14 items) and parents (18 items) [22]. A total score of ≥ 1.5 SD above the mean of the Dutch reference group, depending on age and gender, indicates possible executive function deficits and was considered abnormal. A total score between > 1 and < 1.5 SD above the mean of the Dutch reference group was considered borderline abnormal [22]. A (borderline) abnormal score would be an indication to refer for an assessment of executive functioning of the child by a professional.

#### 2.2.4 Behaviour

Behaviour was assessed by the Dutch version of the Strength and Difficulties Questionnaire (SDQ) and the Strengths and Weaknesses of ADHD symptoms and Normal behaviour (SWAN) questionnaire.

Children filled out the one‐sided self‐rated SDQ for 11–17 year olds (25 items). Parents and teachers filled out the one‐sided SDQ for parents or teachers of 4–17 year olds (25 items). A Total Difficulties Score above the 90th percentile of the Dutch reference group, depending on age and gender, was considered abnormal for all informants [23, 24]. A Total Difficulties Score between 80 and 90th percentile was considered borderline abnormal.

Parents filled out the SWAN questionnaire (18 items). An abnormal score was considered when the summary score was above the 95th percentile (i.e., 1.65 SD above the mean based on population data presented in earlier publications with the following cut‐offs; ADHD‐Combined summary score 2.11, ADHD‐Inattention Deficit 2.48, ADHD‐Hyperactivity/Impulsivity 2.00) [25, 26].

#### 2.2.5 Composite Outcome at 14 Year's Follow Up

We used a composite of abnormal child cognitive and/or behaviour questionnaire scores to identify an abnormal developmental outcome for all children in the follow‐up. We considered an abnormal developmental outcome if at least one of the following applied:

− Attending special education at the time of follow‐up;
− Scores on the parental or self‐reported BRIEF screener of ≥ 1.5 SD
− Scores on the parental, child, or teacher's SDQ of > 90th percentile.

#### 2.2.6 Gender Identity

Parents filled out 16 items about child's gender‐typed behaviour in the Gender Identity Questionnaire (GIQ). Lower scores indicated more cross‐gendered behaviour according to the DSM‐IV criteria for Gender Identity Disorder (GID) [27]. We used a Dutch validation study that defined a mean score of 2.21 or lower (0.47 SD, range 1.00–5.00) and 2.66 or lower (0.64 SD, range 1.00–5.00) to identify the number of children that meet criteria for GID or were subthreshold for GID, respectively [28].

#### 2.2.7 General Health

Children's current and previous health related problems were assessed through a general health questionnaire to be answered by parents [29, 30, 31]. We categorised the need for children's (para)medical care, previous hospital admissions and/or need for surgery and medication used in the past 12 months. Information about breastfeeding in the first 6 months of life and sociodemographics of children and parents (e.g., educational levels and family structure) were collected as well.

### 2.3 Statistical Analysis

Analysis was performed based on the intention‐to treat principle and sample size was calculated as described in the study protocol [18].

We compared baseline characteristics of participants allocated to the 17‐OHPC and placebo, and between follow‐up participants and those who were lost to follow‐up using unpaired *t*‐test, Mann Whitney *U* test, Chi‐squared test or Fisher's exact test as appropriate. Comparisons in follow‐up participants, between 17‐OHPC and placebo group, were made using linear regression models for continuous outcomes and logistic regression models for dichotomous outcomes. All models estimated robust standard errors clustered on mother (sandwich‐type SEs) to take into account the twin pregnancy structure of our data. We also adjusted for the following confounders: maternal education, age at follow‐up, parents smoking at follow‐up, gestational age at birth and ethnicity. We used inverse probability weighting (IPW) to fit a propensity score based on these confounders using restricted cubic splines for age and gestational age (5 knots). We used this model to estimate stabilised weights and truncated the weights at the bottom and top 0.5%. We assessed balance between groups in terms of standardised mean difference (SMD) after weighting. Finally, we again fitted the above linear or logistic regression models with cluster robust standard errors and applied the weights. IPW analysis was not possible for the outcome educational attainment. Record linkage with the education registries of Statistics Netherlands did not allow us to link this with all required characteristics of participants in the study. Outcomes based on record linkage were adjusted for twin pregnancy and maternal education.

Sensitivity and subgroup analyses were defined a priori and performed to give insight into the robustness of the results [18]. The number of children evaluated that scored abnormal in all questionnaires per outcome (e.g., for the composite cognition, it will be defined as an abnormal score in the BRIEF screener self‐ and parental report). All analyses were performed using IBM SPSS Statistics, version 28. SPSS version 25 was used within the microdata environment of Statistics Netherlands.

## 3 Results

**FIGURE 1:**
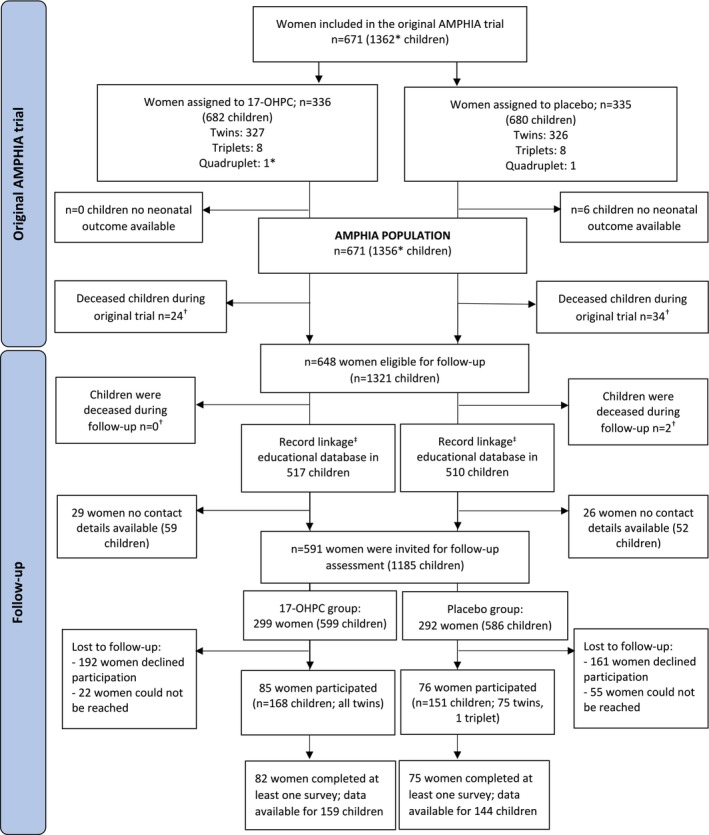
Flowchart of participants in the AMPHIA follow‐up. 17‐OHPC, 17a‐hydroxyprogesterone caproate. *In the original AMPHIA trial, a quadruplet was documented as triplet. †In the original AMPHIA trial, one child was incorrectly documented as deceased in the 17‐OHPC group. Deceased children were included in this follow‐up to calculate total mortality. ‡Record linkage with the educational database of Statistics Netherlands.

In the AMPHIA trial 671 women gave birth to 1356 children. At the time of follow‐up, 60 children were deceased (*n* = 58 perinatal and *n* = 2 child deaths until 14 years of age). The total mortality until 14 years was 24 (3.7%) in the 17‐OHPC group versus 36 (5.6%) in the placebo group (OR 0.75 (0.36–1.53)), Figure 1 and Table 2.

Information of 1027 of 1296 (79%) surviving children could be linked with the CBS data (Figure 1). One of the education outcomes, the CITO scores, was available for 293 children (23%). The characteristics of these 293 children (birth weight, gestational age, sex, multiplicity) were not statistically different compared to the 734 children without CITO outcomes (Table S1).

Developmental outcomes, as assessed by questionnaires, were obtained for 303 of 1296 (23%) surviving children of whom 159 in 17‐OHPC and 144 in the placebo group (Figure 1). A total of 157 parents, 267 children and 202 teachers completed the questionnaires. Compared to the original AMPHIA trial, more participants of European origin and more participants with a high level of education participated in this follow‐up. The proportion of women who smoked during pregnancy was lower among participants in follow‐up. Fewer children born with a birthweight under 1500 g participated in the follow‐up study (Table 1). Baseline characteristics of women and children in the 17‐OHPC and placebo group participating in this follow‐up are shown in Table 1 and Table S2. The median age of the children at follow‐up was 13.2 years (IQR 12.8 to 13.8), with the youngest and oldest child being 11.5 and 14.9 years respectively. Participating women allocated to placebo had completed a higher level of education at study entry of the original AMPHIA trial than those in the 17‐OHPC group (75% vs. 52%).

**TABLE 1:** Baseline characteristics of women and their child(ren) participating in the follow‐up and those that were lost to follow‐up.

Maternal level—Characteristics at entry at the original AMPHIA trial and pregnancy outcomes	Participants with at least one completed questionnaire *n* = 157 (303 children)				AMPHIA trial population *n* = 671 (1356 children)			
	n/n^a^	17‐OHPC *n* = 75	Placebo *n* = 82	Risk estimate (95% CI)	n/n^b^	Follow‐up *n* = 157	Lost to follow‐up *n* = 514	Risk estimate (95% CI)
Maternal age at randomisation, mean (SD)	82/75	33.2 (4.0)	33.5 (4.2)	−0.33 (−1.62–0.95)	157/514	33.4 (4.0)	32.6 (4.7)	−0.80 (−1.62–0.02)
Nulliparity, *n* (%)	82/75	42 (51.2)	43 (57.3)	0.78 (0.42–1.47)	157/514	85 (54.1)	271 (52.7)	1.06 (0.74–1.52)
Smoking during pregnancy, *n* (%)	80/72	4 (5.0)	2 (2.8)	1.84 (0.33–10.37)	152/509	6 (3.9)	57 (11.2)	0.33 (0.14–0.77)
BMI, median (IQR)	72/78	23.8 (20.9–26.5)	23.1 (21.2–25.4)	−0.55 (−1.79–0.67)	150/444	23.4 (21.1–26.1)	23.6 (21.1–26.6)	0.24 (−0.48–0.94)
Higher maternal education^c^, *n* (%)	65/60	34 (52.3)	45 (75.0)	0.37 (0.17–0.78)	125/370	79 (63.2)	163 (44.1)	2.18 (1.44–3.31)
Ethnic origin European, *n* (%)	81/73	77 (95.1)	70 (95.9)	0.83 (0.18–3.82)	154/493	147 (95.5)	428 (86.8)	3.19 (1.43–7.11)
Fertility treatment^d^	81/75	36 (44.4)	33 (44.0)	1.02 (0.54–1.92)	156/514	69 (44.2)	191 (37.2)	1.34 (0.93–1.93)
Triplet or Quadruplet, *n* (%)	82/75	0	1 (1.3)	0.99 (0.96–1.01)	157/514	1 (0.6)	17 (3.3)	5.34 (0.70–40.42)
Monochorionic, *n* (%)	82/75	14 (17.1)	10 (13.3)	0.75 (0.31–1.80)	157/514	24 (15.3)	90 (17.5)	1.18 (0.72–1.92)
PPROM, *n* (%)	69/66	9 (13.0)	10 (15.2)	0.84 (0.32–2.22)	135/458	19 (14.1)	43 (9.4)	1.58 (0.89–2.82)
Corticosteroids, *n* (%)	82/75	20 (24.4)	19 (25.3)	0.95 (0.46–1.96)	157/512	39 (24.8)	145 (28.3)	0.84 (0.56–1.26)
Pregnancy duration in weeks^e^ < 28 weeks, *n* (%)	82/75	1 (1.2)	0	0.84 (0.45–1.57)	157/510	1 (0.6)	36 (7.1)	0.80 (0.56–1.14)
Between 28 + 0 and 31 + 6 weeks, *n* (%)		8 (9.8)	4 (5.3)			12 (7.6)	33 (6.5)	
Between 32 + 0 and 36 + 6 weeks, *n* (%)		29 (35.4)	34 (45.3)			63 (40.1)	207 (40.6)	
< 37 weeks (all), *n* (%)		38 (46.3)	38 (50.7)			76 (48.4)	276 (54.1)	

Child level – characteristics and outcomes of original AMPHIA trial	n/n^a^	17‐OHPC *n* = 159	Placebo *n* = 144	Risk estimate (95% CI)	n/n^b^	Follow‐up *n* = 303	Lost to follow‐up *n* = 1053	Risk estimate (95% CI)
Composite adverse outcome of the AMPHIA trial, *n* (%)^f^	159/144	19 (11.9)	13 (9.0)	1.37 (0.65–2.88)	303/1052	32 (10.6)	158 (15.0)	0.67 (0.45–1.00)
Birthweight < 1500 g, *n* (%)	159/144	11 (6.9)	5 (3.5)	2.07 (0.70–6.10)	303/1044	16 (5.3)	138 (13.2)	0.37 (0.21–0.63)
Male sex *n* (%)	159/144	83 (52.2)	62 (43.1)	1.44 (0.92–2.27)	303/1033	158 (52.1)	507 (49.1)	0.89 (0.69–1.14)

Abbreviation: n/a, not applicable.

^a^ Number of analysed participants without missing data. 17‐OHPC/placebo group.

^b^ Number of analysed participants without missing data. Follow‐up/lost to follow‐up.

^c^ Higher vocational education and university education.

^d^ Pregnant after ovarian hyperstimulation, in vitro fertilisation, or intracytoplasmic sperm injection.

^e^ < 37 weeks compared to ≥ 37 weeks of gestation.

^f^ Children in the AMPHIA trial who were diagnosed by a neonatologist with severe respiratory distress syndrome, bronchopulmonary dysplasia, intraventricular haemorrhage grade IIB or worse, necrotizing enterocolitis, proven sepsis, or death before discharge from the hospital.

After weighting, the SMD for the difference between groups of all five confounders was below 0.10, indicating that groups were balanced in terms of those confounders. The average stabilised weight was 1.00 in both groups (25th–75th percentile: 0.73–1.13).

### 3.1 Educational Attainment

From all AMPHIA children that could be linked with the CBS data (*n* = 1027, 517 in 17‐OHPC and 510 in placebo group), the number of children attending special education at primary school age did not significantly differ between groups (4.1% vs. 5.5%; aOR 0.74 95% CI 0.41–1.33). There was also no difference in the teacher's advice for vocational secondary education at age 12 (63.2% vs. 59.2%; aOR 1.17 95% CI 0.90–1.53), Table 2. The CITO scores of a subgroup of children (*n* = 293, 144 in 17‐OHPC and 149 in placebo group) did not significantly differ between groups (adjusted mean difference −0.43, 95% CI −2.50 to 1.65), Table 2.

**TABLE 2:** Outcomes of child mortality, educational attainment, cognition, behaviour and gender identity at 11–14 years of age in the 17‐OHPC group compared to placebo.

Data from record linkage^a^					
	*n* = 1356 children (n/n^b^)	17‐OHPC *n* = 682	Placebo *n* = 674	Crude OR or mean difference (95% CI)^c^	OR after IPW or adjusted mean difference (95% CI)^d^
*Mortality*					
Mortality until 14 years^j^, *n* (%)	1285 (643/642)	24 (3.7)	36 (5.6)	0.65 (0.32–1.31)	0.75 (0.36–1.53)
*Educational attainment—primary school*					
Special education, *n* (%)	1027 (517/510)	21 (4.1)	28 (5.5)	0.73 (0.41–1.30)	0.73 (0.41–1.33)
Teacher's secondary school advice, *n* (%)					
Vocational education advice	1027 (517/510)	327 (63.2)	302 (59.2)	1.19 (0.92–1.53)	1.17 (0.90–1.53)
Vocational education advice (excluding special education)	978 (496/482)	306 (61.7)	274 (56.8)	1.22 (0.95–1.58)	1.21 (0.93–1.58)
Higher secondary school level	1027 (517/510)	190 (36.8)	208 (40.8)	0.84 (0.66–1.08)	0.85 (0.66–1.11)
CITO test scores^k^, mean (SD)	293 (144/149)	535.5 (9.1)	536.4 (9.7)	−0.95 (−3.10–1.21)	−0.43 (−2.50–1.65)

Data from self‐reported, parental and teacher questionnaires					
	*n* = 303 children (n/n^b^)	17‐OHPC *n* = 159	Placebo *n* = 144	Crude OR (95% CI)^c^	OR after IPW (95% CI)^d^
*Educational attainment – secondary school*					
Current level of education, *n* (%)					
Special education	289 (151/138)	4 (2.6)	6 (4.3)	0.60 (0.12–2.99)	0.54 (0.09–3.24)
Vocational secondary education	289 (151/138)	61 (40.4)	42 (30.4)		
Higher secondary school level	289 (151/138)	86 (57.0)	90 (65.2)		
*Cognition*					
Abnormal BRIEF screener^e^, *n* (%)	292 (151/141)	11 (7.3)	14 (9.9)	0.71 (0.30–1.72)	0.90 (0.35–2.31)
Borderline abnormal BRIEF screener^e^, *n* (%)	292 (151/141)	36 (23.8)	23 (16.3)	1.61 (0.89–2.90)	1.68 (0.80–3.53)
*Behaviour*					
Abnormal SDQ^f^, *n* (%)	301 (158/143)	33 (20.9)	26 (18.2)	1.19 (0.62–2.26)	1.31 (0.68–2.53)
Borderline abnormal SDQ^f^, *n* (%)	301 (158/143)	23 (14.6)	18 (12.6)	1.18 (0.62–2.28)	0.92 (0.46–1.86)
Abnormal SWAN^g^, *n* (%)	257 (135/122)	8 (5.9)	4 (3.3)	1.86 (0.42–8.29)	1.76 (0.41–7.63)
*Gender identity*					
Meet criteria for GID^h^, *n* (%)	257 (135/122)	0	0	n/a	n/a
Subthreshold for GID^h^, *n* (%)	257 (135/122)	1 (0.7)	3 (2.5)	0.30 (0.03–2.87)	0.22 (0.02–2.22)
*Composite outcome*					
Composite of abnormal child development^i^, *n* (%)	303 (159/144)	37 (23.3)	29 (20.1)	1.20 (0.65–2.23)	1.31 (0.66–2.56)

^a^ The Dutch Personal Records Database were checked for the possible occurrence of child death and educational attainment at primary school was obtained from the educational databases of Statistics Netherlands (CBS).

^b^ Number of analysed children without missing data (17‐OHPC/placebo group).

^c^ 95% CI adjusted for a twin pregnancy using robust standard errors clustered on mother. For the outcome “CITO scores” a mean difference adjusted for a twin pregnancy is presented.

^d^ OR adjusted for a twin pregnancy and the confounders maternal education, age at follow‐up, parents smoking at follow‐up, gestational age at birth and ethnicity. For the outcome “CITO scores” an adjusted mean difference adjusted for a twin pregnancy and maternal education is presented.

^e^ BRIEF: Behaviour Rating Inventory of Executive Function. Reported by parents/caregivers or self‐report. Scores are mutually exclusive. A score of ≥ 1.5 SD above the mean of the Dutch reference group was considered abnormal. A score of > 1 and < 1.5 SD above the mean of the Dutch reference group was considered borderline abnormal.

^f^ SDQ: Strength and Difficulties Questionnaire. Reported by parents/caregivers or child or teacher. Scores are mutually exclusive. Scores > 90th percentile of the Dutch reference group were considered abnormal. A borderline abnormal score was reported if scores were between 80 and 90th percentile of the Dutch population.

^g^ SWAN; Strengths and Weaknesses of ADHD symptoms and Normal behaviour questionnaire. Scores reported by parents/caregivers. Abnormal scores were defined as > 1.65 SD above the mean of the reference group.

^h^ GID, Gender Identity Disorder. Scores were reported by parents/caregivers. Children with ≤ 2.21 points meet criteria for GID and children with scores between 2.21 and 2.66 points were considered subthreshold for GID.

^i^ Composite of abnormal child development: abnormal executive functioning according to the parental or self‐reported BRIEF screener or attending special education or presence of behavioural problems according to the parental or child or teacher's SDQ.

^j^ Of all deceased children, *n* = 58 died perinatally (from 16 weeks of gestation up to discharge from the hospital) and *n* = 2 child deaths occurred.

^k^ Most frequently used standardised primary school leaving assessment test (Centraal Instituut voor Toets Ontwikkeling [CITO]).

In the follow‐up assessment, information about school attainment was collected for 289 children (151 in 17‐OHPC and 138 in placebo group). All children except one child in the placebo group, attended secondary school of whom 10/289 (3.5%) children attended special education (Table 2). In total, 101 children had need for extra help in‐ or outside the classroom (52/159 (32.7%) in the 17‐OHPC group and 49/141 (35.0%) in the placebo group; the aOR was 0.97 95% CI 0.57 to 1.64). The number of children that repeated a grade was 23/151 (15.2%) in the 17‐OHPC and 17/137 (12.4%) in the placebo group, with an aOR 0.83 95% CI (0.30–2.25).

### 3.2 Cognition

The self‐ and parental report of the BRIEF screener was filled out for 267 and 286 children respectively (21% and 22% of all surviving children). Although not significant, the number of children with an abnormal BRIEF‐screener was lower in the 17‐OPHC group (7.3% vs. 9.9%, OR 0.90 95% CI 0.35–2.31); Table 2 and Table S3. Sensitivity analyses showed that three children had an abnormal outcome in both self‐report and parental report (2/139, 1.4% in the 17‐OHPC group and 1/122, 0.8% in the placebo group). The aOR was 0.69 (95% CI 0.09–4.54).

### 3.3 Behaviour

The SDQ self‐report was filled out by 267 children, the parental report for 285 children and the SDQ teacher report for 202 children (21, 22% and 16% of all surviving children, respectively). No differences were observed regarding abnormal SDQ scores in the 17‐OHPC and placebo group (20.9% vs. 18.2%; OR 1.31 95% CI 0.68–2.53), nor in the Total Difficulties Scores (Table 2 and Table S3). The SWAN questionnaire was filled out for 257 children (20% of all surviving children), and results also did not differ between groups (Table 2 and Table S3). Sensitivity analyses showed no children with abnormal scores in all SDQ reports and the SWAN questionnaire.

### 3.4 Composite Outcome at 14 Year's Follow Up

The composite outcome of abnormal child development during follow‐up was observed in 37/159 (23.3%) in 17‐OHPC and in 29/144 (20.1%) in the placebo group (OR 1.31, 95% CI 0.66–2.56).

### 3.5 Gender Identity

None of the follow‐up participants met the criteria for GID (Table 2).

### 3.6 General Health

Outcomes regarding children's general health (as reported by their caregivers) are shown in Table S4. No differences were seen between the 17‐OHPC and placebo groups in respect to neurological or behavioural disorders, medical diagnoses, diseases or impairments, admissions to the hospital and medication use.

## 4 Discussion

### 4.1 Main Findings

In this follow‐up study among twins, we observed no effects of prenatal 17‐OHPC versus placebo on child survival, development or health till age 14 years. Specifically, we found no differences regarding children's mortality, educational attainment, cognitive and behavioural outcomes. For some other outcomes there is not enough data to support conclusion.

### 4.2 Strengths and Limitations

Our study has several strengths and limitations to report. A first strength of our study is the use of two follow up strategies; data linkage and questionnaires on a broad array of outcomes to present the full scope of long‐term developmental effects of prenatal progestagen exposure. Accordingly, we were able to give an accurate overview on child's survival up to 14 years of age and collect important information regarding educational attainment. Second, multiple informants (i.e., parents, teachers and the children themselves) were used to acquire information on different domains of functioning for which validated questionnaires for the Dutch population were used. Consequently, we were able to obtain a comprehensive overview of child development, and the risk of bias was reduced. Third, this study was the first to assess the effect of progestagens on the outcome regarding gender identity and had a follow‐up until early adolescence. Last, this study adds to the number of follow‐up studies after RCTs evaluating the long‐term safety of interventions performed to prevent preterm birth [32].

A first limitation of this study is the high loss to follow‐up in this study. We included 27% of the original AMPHIA trial participants (i.e., 23% of all surviving children born in the original trial). However, due to the CBS micro data environment availability of national data at the age of 12 years as a result of data linkage this low follow up rate is counterbalanced. Thereby, the loss did not impact our power to measure potential differences between the groups according to our precalculated sample size, but it does increase the potential for selection bias [18]. With the use of record linkage we obtained information on 79% of the surviving children, which strengthens the validity of our findings derived from the questionnaires. Second, bias could be induced by the fact that baseline characteristics in our study were different between our two comparison groups; compared to the 17‐OHPC group, more women with a higher educational level at randomisation participated in the placebo group. However, since this study mainly focussed on evaluating the chances of long‐term harm to the children exposed with 17‐OHPC in utero, the fact that more women in the placebo group had higher educational attainment, should not favour effects of 17‐OHPC. Most importantly, the outcomes did not significantly change after adjustment for maternal education. Third, compared to the original AMPHIA trial, fewer women with low social economic status, non‐European ethnicity and women who smoked participated in the follow up study. In addition, fewer children with a birthweight below 1500 g were assessed in this study. Low social economic status, non‐European ethnicity, smoking and low birthweight are all associated with poorer pregnancy and child outcomes and, therefore, hamper generalizability of our results [33, 34, 35, 36].

### 4.3 Interpretation

The results are in line with a systematic review reporting no harmful effects of prenatal progesterone exposure on child development up to 8 years [15]. Our study shows the great potential of two additive follow up strategies, using both readily available data from school registries as well as in depth questionnaires.

Progestagens are used in clinical practice, for example in preterm birth prevention. Mechanisms of action may differ between the different types of progestagens [37]. The most recommended type of progestagen in present‐day obstetrics is the natural vaginal progesterone [38]. Although the effect on preterm birth prevention is similar for vaginal (natural) progesterone and 17‐OHPC, and therefore the impact on long term outcomes, differences in pharmacokinetics exist [39]. Where vaginal (natural) progesterone is being converted to the neuroprotective metabolite allopregnanolone, this is slightly different for 17‐OHPC. In theory, this neuroprotective metabolite could benefit child outcomes, resulting in better child outcomes by vaginal (natural) progesterone compared to 17‐OHPC. Given the lack of association between vaginal (natural) progesterone and 17‐OHPC in our study, we speculate that exposure to vaginal (natural) progesterone, like 17‐OHPC, is unlikely to have any harmful effects on child outcomes [1]. Our findings for 17‐OHPC are however not fully generalisable to the use of other types of progestogens.

In the original AMPHIA trial, children were exposed to 17‐OHPC in the second and third trimester of pregnancy. Progestagens are also used for purposes in the first trimester (e.g., in artificial reproductive technologies). Animal studies show that progesterone receptors are ubiquitously expressed in the developing fetal brain [4, 11]. It is therefore possible that fetal development could be differentially affected if progesterone treatment would start earlier in pregnancy.

## 5 Conclusion

In offspring of women with a multiple pregnancy, the use of 17‐OHPC in pregnancy is unlikely to provide positive or negative effects on child outcome related to mortality, educational attainment, cognition and behaviour. Future studies should focus on the effect on children's outcomes after prenatal exposure to natural progesterone and use of progestagens in the first trimester.

## Author Contributions

E.V.J.L.S., M.A.B., A.G.L., M.J.J.F., C.S.H.A.‐M., A.L.B., N.E.S., R.C.P., E.P., M.A.O. and J.H. were involved in conception and design of the study. Data was obtained by E.V.J.L.S., A.C.J.R. and A.C.L. and analyses were performed by R.E., E.V.J.L.S. and A.C.J.R. The first draft of the article was made by E.V.J.L.S., M.A.B., A.C.J.R. and J.H. and all other authors reviewed and revised the article. All authors approved the final version of the article for publication.

## Funding

This research was funded by the Amsterdam Reproduction and Development research institute (AR&D). Grant number: 2019.02.003.

## Ethics Statement

The study was approved by the Medical Research Ethics Committee of the Amsterdam University Medical Center (Amsterdam UMC), location AMC (W20_234#20.268).

## Conflicts of Interest

B.W.M. is supported by a NHMRC Investigator grant (GNT1176437). B.W.M. reports consultancy, travel support and research funding from Merck and consultancy for Organon and Norgine. All other authors declare no conflicts of interest.

## Supporting information


**Table S1:** Characteristics of children with and without CITO outcomes in education registry Statistics Netherlands.
**Table S2:** Baseline characteristics of women and their child(ren) participating in the follow‐up.
**Table S3:** Continuous outcome measures of child cognition and behaviour at 11–14 years of age in the 17‐OHPC group compared to placebo.
**Table S4:** Current and previous health status of children participating in the follow‐up between 17‐OHPC and placebo group.

## Data Availability

The data that support the findings of this study are available from the corresponding author upon reasonable request.
