# Supplementary material for: Prenatal 17‐Alpha‐Hydroxyprogesterone Caproate Exposure in Twins and Childhood Outcomes: 14‐Year Follow‐Up of a Randomised Trial
Source: BJOG. 2026 Mar 23;133(9):1742–52. doi: 10.1111/1471-0528.70223 (PMC13419107; doi:10.1111/1471-0528.70223)
Supplement: Supplementary file 1 — Table S1: Characteristics of children with and without CITO outcomes in education registry Statistics Netherlands. Table S2: Baseline characteristics of women and their child(ren) participating in the follow‐up. Table S3: Continuous outcome measures of child cognition and behaviour at 11–14 years of age in the 17‐OHPC group compared to placebo. Table S4: Current and previous health status of children participating in the follow‐up between 17‐OHPC and placebo group. [file BJO-133-1742-s001.docx]

**Table S1.** Characteristics of children with and without CITO outcomes in education registry Statistics Netherlands.

|  | **n=1027 children**  **(n/n)*** | **CITO score available n=293** | **No CITO score**  **available n=734** | **p-value** |
| --- | --- | --- | --- | --- |
| Birth weight, mean (SD) | 1025 (292/733) | 2437 (618) | 2407 (608) | 0.48 |
| Gestational age at birth in weeks, mean (SD) | 1019 (289/730) | 35.7 (2.7) | 35.5 (3.0) | 0.31 |
| Male sex, n(%) | 1027 (293/734) | 145 (49.5) | 380 (51.8) | 0.51 |
| Multiplicity^†^, n(%) | 1027 (293/734) | 147 (50.2) | 369 (50.3) | 0.98 |

CITO: most frequently used standardized primary school leaving assessment test (Centraal Instituut voor Toets Ontwikkeling [CITO]).
*number of analyzed participants without missing data. CITO score available / No CITO score available.
†first delivered infant.

**Table S2.** Baseline characteristics of women and their child(ren) participating in the follow-up.

| **Child level – characteristics at follow-up** | **n/n*** | **17-OHPC n=159** | **Placebo n=144** | **P-value** |
| --- | --- | --- | --- | --- |
| Child age at follow-up, median (IQR) | 159/144 | 13.2 (12.8-13.8) | 13.3 (12.9-13.8) | 0.19 |
| Social background at follow-up  - Two-parent family^¶^, n(%) - Twin is the oldest of the siblings, n(% - Primary language Dutch, n(%) - Bilingual, n(%) - Breastfeeded at least 6 months^#^, n(%) - ≥1 parent born outside the Netherlands, n(%) - (Both) parents completed high professional education†, n(%) - Smoking in or outside the house, n(%) | 159/144 150/139 155/143 155/143 116/103 134/128 154/141  155/143 | 139 (87.4) 64 (42.7) 144 (92.9) 74 (47.7) 37 (31.9) 14 (10.4) 106 (68.8)  18 (11.6) | 124 (86.1) 68 (48.9) 140 (97.9) 71 (49.7) 22 (21.4) 12 (9.4) 102 (72.3)  14 (9.8) | 0.74 0.29 0.04 0.74 0.08 0.77 0.51  0.61 |

* number of analyzed participants without missing data. 17-OHPC / placebo group.
¶ Living in a two-parents household: children living with two (biological) parents or one (biological) parent with de facto relationship.
#Children who were breastfeed for at least the first 6 months of life with or without infant formula.
† higher vocational education and university education.

**Table S3.** Continuous outcome measures of child cognition and behavior at 11-14 years of age in the 17-OHPC group compared to placebo.

|  | **n=303 children**  **(n/n)*** | **17-OHPC n=168** | **Placebo n=151** | **Curde mean difference (95% CI)**† | **Mean difference after IPW (95% CI)**‡ |
| --- | --- | --- | --- | --- | --- |
| **Cognition** |  |  |  |  |  |
| BRIEF screener parental report (Total Score Executive Function), mean (SD) | 286 (136/150) | 30.23 (6.80) | 29.59 (6.70) | 0.64 (-0.93 to 2.44) | 1.29 (-0.69 to 3.27) |
| BRIEF screener self-report (Total Score Executive Function), mean (SD) | 267 (127/140) | 22.18 (3.94) | 21.25 (4.29) | 0.93 (-0.19 to 2.05) | 0.82 (-0.37 to 2.01) |
| **Behavior** |  |  |  |  |  |
| SDQ parental report (Total Difficulties Score), mean (SD) | 136/149 | 7.11 (5.03) | 6.73 (5.05) | 0.38 (-1.05 to 1.81) | 0.57 (-0.95 to 2.08) |
| SDQ self-report (Total Difficulties Score), mean (SD) | 127/140 | 10.14 (5.75) | 8.88 (5.40) | 1.25 (-0.35 to 2.86) | 1.31 (-0.42 to 3.04) |
| SDQ teacher’s report (Total Difficulties Score), mean (SD) | 93/109 | 5.59 (4.87) | 4.99 (4.66) | 0.60 (-0.81 to 2.01) | 0.63 (-0.87 to 2.13) |
| SWAN parental report, mean (SD) - ADHD-Inattention Deficit - ADHD-Hyperactivity/Impulsivity - ADHD-Combined summary score | 122/135 | 0.42 (0.91) 0.51 (0.89) 0.47 (0.82) | 0.39 (0.88) 0.49 (0.87) 0.44 (0.80) | 0.03 (-0.21 to 0.26)  0.02 (-0.24 to 0.27)  0.02 (-0.21 to 0.25) | 0.05 (-0.17 to 0.27)   1. (-0.26 to 0.26)   0.02 (-0.20 to 0.25) |
| **Gender identity** |  |  |  |  |  |
| Child gender identity parental report, mean (SD) | 122/135 | 3.63 (0.39) | 3.53 (0.36) | 0.10 (0.00-0.21) | 0.12 (0.01-0.24) |

* number of analyzed children without missing data (17-OHPC/placebo group).

† Mean difference adjusted for twin pregnancy using robust standard errors clustered on mother.
‡ Mean difference adjusted for twin pregnancy and confounders. maternal education, age at follow-up, parents smoking at follow-up, gestational age at birth and ethnicity.

**Table S4.** Current and previous health status of children participating in the follow-up between 17-OHPC and placebo group.

|  | **n=290 children (n/n)*** | **17-OHPC n=151** | **Placebo n=139** | **Crude OR (95% CI)**** | **OR after IPW**  **(95% CI)***** |
| --- | --- | --- | --- | --- | --- |
| Neurologic or motor disorders†, n(%) | 290 (139/151) | 9 (6.0) | 9 (6.5) | 0.92 (0.31-2.72) | 1.11 (0.36-3.42) |
| Lung disease‡ , n(%) | 290 (139/151) | 16 (10.6) | 13 (9.4) | 1.15 (0.48-2.75) | 1.10 (0.43-2.84) |
| Behavioral disorders^§^, n(%) | 290 (139/151) | 33 (21.9) | 28 (20.1) | 1.11 (0.56-2.11) | 1.13 (0.52-2.46) |
| Visual and hearing impairments, n(%) | 290 (139/151) | 3 (2.0) | 2 (1.4) | 1.39 (0.23-8.35) | 1.16 (0.18-7.35) |
| Allergic and auto-immune disease, n(%) | 290 (139/151) | 7 (4.6) | 9 (6.5) | 0.70 (0.22-2.27) | 0.59 (0.18-1.97) |
| ≥3 admissions to hospital, n(%) | 290 (139/151) | 20 (13.2) | 17 (12.2) | 1.10 (0.49-2.44) | 0.91 (0.40-2.09) |
| ≥3 surgeries, n(%) | 290 (139/151) | 9 (6.0) | 5 (3.6) | 1.70 (0.54-5.35) | 1.25 (0.37-4.31) |
| Medication used in the past 12 months, n(%) - antibiotics  - laxatives  - lung medication  - for eczema  - growth hormone  - for ADHD  - for epilepsy  ≥1 medications, n(%) ≥3 medications, n(%) | 289 (138/151) | 5 (3.3) 10 (6.6) 13 (8.6) 20 (13.2) 0 7 (4.6) 1 (0.7)  44 (29.1) 0 | 2 (1.4) 4 (2.9) 8 (5.8) 17 (12.3) 1 (0.7) 3 (2.2) 0  29 (21.0) 1 (0.7) | 1.55 (0,85-2.82) | (1.69 (0.90-3.19) |
| Problems with motor, language, speech or school function in the past 12 months^\|\|^, n(%) | 290 (139/151) | 13 (8.6) | 12 (8.6) | 1.00 (0.37-2.69) | 1.11 (0.40-3.10) |

* number of analyzed children without missing data (17-OHPC/placebo group).

** OR adjusted for twin pregnancy using robust standard errors clustered on mother

*** OR adjusted for twin pregnancy and confounders maternal education, age at follow-up, parents smoking at follow-up, gestational age at birth and ethnicity.

† Cerebral palsy, epilepsy, other (i.e. traumatic brain injury, migraine, problems in language/speech development, hypermobility, scoliosis).
‡ asthma, bronchitis, CARA or other
§ Autism / Autism spectrum disorder, ADD, ADHD, dyslexia, dyscalculia, depression/anxiety, eating disorder, other psychiatric diagnosis.
|| Disorder in motor development, language/speech development, problems with learning in school.
